# Balanced biogeographic and local environmental effects determine the patterns of microbial diversity in biocrusts at multi-scales

**DOI:** 10.3389/fmicb.2023.1284864

**Published:** 2023-11-09

**Authors:** Yuanlong Li, Fengdi Wang, Haijian Yang, Hua Li, Chunxiang Hu

**Affiliations:** ^1^Hunan Provincial Key Laboratory of Carbon Neutrality and Intelligent Energy, School of Resource and Environment, Hunan University of Technology and Business, Changsha, China; ^2^Key Laboratory of Algal Biology, Institute of Hydrobiology, Chinese Academy of Sciences, Wuhan, China; ^3^Institute of Hematology, Union Hospital, Tongji Medical College, Huazhong University of Science and Technology, Wuhan, China

**Keywords:** biogeography, biological soil crusts, cyanobacteria, microbial diversity, multiple spatial scales, primary succession, species turnover

## Abstract

**Introduction:**

Biodiversity maintenance and its underlying mechanisms are central issues of ecology. However, predicting the composition turnovers of microbial communities at multiple spatial scales remains greatly challenging because they are obscured by the inconsistent impacts of climatic and local edaphic conditions on the assembly process.

**Methods:**

Based on the Illumina MiSeq 16S/18S rRNA sequencing technology, we investigated soil bacterial and eukaryotic communities in biocrusts with different successional levels at a subcontinental scale of Northern China.

**Results:**

Results showed that irrespective of spatial scale, bacterial α diversity increased but eukaryotic diversity decreased with the primary succession, whereas both β diversities decreased at the subcontinental scale compared with smaller scales, indicating that the biogeographic pattern of soil microorganisms was balanced by successional convergence and distance decay effect. We found that the convergence of bacterial and eukaryotic communities was attributed to the turnovers of generalist and specialist species, respectively. In this process, edaphic and climatic factors showed unique roles in the changes of diversity at local/subcontinental scales. Moreover, the taxonomic diversity tended to be more susceptible to climatic and edaphic conditions, while biotic factors (photosynthesis and pigments) were more important to phylogenetic diversity.

**Conclusion:**

Taken together, our study provided comprehensive insights into understanding the pattern of microbial diversity at multiple spatial scales of drylands.

## 1. Introduction

Efficient restoration of degraded soils is dependent on a sufficient understanding of how soil communities are generated and maintained during succession (Lan et al., 2014). It raises questions about the nature of soil biodiversity changes and the extent to which it is regulated (Miralles et al., 2020). However, exploring the interrelations among biodiversity changes, community structures, and environmental conditions remains a great challenge (Gotelli et al., 2017; Magurran et al., 2018; O'Sullivan et al., 2019). A number of previous studies have demonstrated that species diversity increases with primary succession across a wide range of different habitats (Jessup et al., 2004; Nemergut et al., 2013; Angel Fernandez-Martinez et al., 2017). Meanwhile, the degree of differentiation between communities (i.e., β diversity) at a certain successional stage is expected to decrease with development, which is due to the selection effect under homogeneous environments (Dini-Andreote et al., 2015). However, evidence of these observed characteristics of microbial diversity is primarily achieved at a single spatial scale (Dornelas et al., 2014), which is particularly influenced by local abiotic conditions. It makes the meta-analysis of microbial diversity across individual studies unfeasible and considerably hinders the comprehensive understanding of the mechanism of diversity maintenance in this mega-diverse community. Therefore, an integrated view that depicts the diversity pattern of soil microorganisms at multiple spatial scales and assesses their co-variability with the environments is being proposed increasingly (Fierer et al., 2010; Bracken et al., 2017).

As a model system in a community (Bowker et al., 2014), biological soil crusts (biocrusts) dominate the topsoil of drylands worldwide and represent a crucial functional component of the ecosystem (Rossi et al., 2017; Li et al., 2021; Weber et al., 2022). According to the edaphic properties of development and community compositions, biocrusts can be classified into different successional stages (Lan et al., 2013). Distinct sets of microbial members reportedly constitute each successional stage of biocrusts, irrespective of geographic separation. The compositions of soil microorganisms in different stages converged at taxonomic and phylogenetic facets at a large spatial distance, whereas various biocrust components with different successional stages can be used to establish a mosaic pattern of micro-landscape at the centimeter scale (Bowker et al., 2013; Weber et al., 2016). Given the combined effect of convergence succession (Xu et al., 2020), high dispersal limitation (Li and Hu, 2021), and special patchy distribution of biocrusts (Li et al., 2016), regional species pools under different environments may contain only closely related species that adapt to the particular habitat of biocrusts. Thus, an emerging question rooted in the primary succession of biocrusts is whether the patterns of biodiversity change are idiosyncratic at local and continental scales, which is associated with the regimes of microbial assembly at multiple spatial scales.

Meanwhile, environmental factors are considered an important cause of the changes in microbial diversity (Rillig et al., 2019). However, the effect of environmental factors on diversity to a large extent is spatially autocorrelated, especially in biocrust communities. In small-scale habitats, biocrusts can tolerate limited moisture and nutrients and respond rapidly to pulsed ambient conditions (Cable and Huxman, 2004; Schwinning and Sala, 2004). A patchy micro-landscape is partly attributed to soil properties, as well as the decomposition of cyanobacterial biomass followed by the releases of exopolysaccharides (EPS), by which carbon sources accumulate unevenly and act as a repository for heterotrophic diversity (Rossi and De Philippis, 2015). At the macro-scale, regional climatic variables such as solar irradiation and precipitation are the most important factors affecting the pattern of microbial diversity on all successional components of biocrusts (Couradeau et al., 2016; Fernandes et al., 2018). Meanwhile, high climatic stresses can impede the succession and rollback of well-developed biocrusts into an early successional stage (Johnson et al., 2012; Reed et al., 2012; Ferrenberg et al., 2015), at which microbial composition significantly changes. Therefore, the heterogeneity of microbial biodiversity in biocrusts can probably be generated through interaction among climatic, edaphic, and biotic drivers. However, the relative importance and uniqueness of these influences on the pattern of microbial biodiversity in biocrusts at different scales remain unclear.

In this study, we performed the analyses on a dataset comprised of cyanobacterial, cyanobacterial-lichen, and moss-dominated crusts from 50 sites across 3,000 km in northern China (Li and Hu, 2021). These components constituted a series of successional stages from the initial to mature biocrusts (Walker et al., 2010; Ladau and Eloe-Fadrosh, 2019). We defined the local and continental scales according to the size of sampling areas (Supplementary Figure S1). Illumina MiSeq 16S/18S rRNA sequencing was used to examine the compositions of bacterial and eukaryotic communities of different successional biocrusts. Then, we further determined the characteristics and dynamics of the associations of climatic, edaphic, and biotic factors with α/β-diversity of microbial communities, to explore the underlying mechanisms of biodiversity maintenance in biocrusts. These analyses allow us to address three questions: First, does a shifting pattern of α and β diversity exist at local and continental scales along succession? Second, is there a balanced biogeographic pattern between bacterial and eukaryotic communities in soils? Third, which factors are more pivotal to underpin the change of microbial diversity in drylands?

## 2. Materials and methods

### 2.1. Sampling, definition of spatial scales, and data collection

35 cyanobacterial, 6 cyanobacteria-lichen, and 9 moss crust-dominated sites were included in a wide range of transects across seven major deserts and the Loess Plateau in northern China [see Li and Hu (2021)]. A total of 140 cyanobacterial, 24 cyanobacteria-lichen, and 36 moss crust samples were collected. Criteria of successional stages were established based on a previous study (Lan et al., 2012), and more details are described in the Supplementary Methods. Biocrusts together with apparently attached subsoil were collected with a shovel and preserved in sterilized plastic Petri dishes to ensure integrity. Macroscopic moss plants were removed from the moss-dominated biocrust to eliminate their potential influence on sequencing and physicochemical measurements.

We defined the *local scale* as the minimum area concurrently covering three successional stages (4 local areas gained) and the *continental scale* as the entire study area (Supplementary Figure S1). The environmental variables were classified into climatic and edaphic factors. Climatic factors included altitude, mean annual precipitation, mean annual sunshine duration, aridity index, windspeed, and mean annual temperature. These data are referenced from the National Meteorological Information Center (http://data.cma.cn/). Edaphic factors included soil texture, crust thickness, water content, soil pH, total phosphorus, total nitrogen, total organic carbon, and salinity. Besides, biotic factors included variable fluorescence/maximal fluorescence, gross photosynthesis/respiration, extracellular polysaccharide, bacteriochlorophyll *a*, scytonemin, chlorophyll *a*, alkaline protease, β-glucosidase, and alkaline phosphatase (see Supplementary Methods).

Total genomic DNA was extracted using the PowerSoil ®DNA Isolation Kit (Mo Bio, Carlsbad, CA, USA). The V3-V4 region of the bacterial 16S rRNA gene was amplified with the primers 338F/806R (Mori et al., 2014), and the V4 region of the eukaryotic 18S rRNA gene was amplified with the primers 3NDF/V4_euk_R2 (Amaral-Zettler et al., 2009). Sequencing was performed on the Illumina MiSeq PE300 platform (Illumina, San Diego, CA, USA). Raw FASTQ files were demultiplexed, quality filtered by Trimmomatic, and merged by FLASH with the following criteria: (i) reads were truncated at any site receiving an average quality score < 20 over a 50 bp sliding window; (ii) primers were exactly matched to allow 2-nucleotide mismatching, and reads containing ambiguous bases were removed; and (iii) sequences with overlap longer than 10 bp were merged according to their overlap sequence. Operational taxonomic units (OTUs, hereafter denoted species) were generated from defined representative sequences, with clustering at 97% similarity. The RDP classifier was used for the taxonomic annotation of representative sequences based on an identity threshold of 0.7 in the SILVA 128 database for bacteria and eukaryotic microorganisms (i.e., fungi, protozoa, and eukaryotic microalgae).

### 2.2. Calculations of biodiversity indices and species turnover

To study the patterns of α and β diversity along with the succession, we calculated the Shannon diversity (*H′*), phylogenetic diversity (PD), species richness, and Pielou evenness (α diversity indices) (Hurlbert, 1971), as well as the Bray-Curtis and weighted-UniFrac dissimilarities between paired samples (β diversity) (Anderson et al., 2006). At the local scale, β diversity was calculated among four adjacent parallel samples within the same successional stage. At the continental scale, the paired difference indices were calculated between the sites several kilometers apart. These analyses were performed by package vegan in *R* (Oksanen et al., 2015). ANOVA was used to analyze the significant level of α and β diversity among three successional stages (SPSS version 20, *p < 0.05, **p < 0.01).

To study the patterns of ubiquitous taxa, we defined the species occurring in >85% of samples as ubiquitous taxa and calculated their relative abundance in each sample. These ubiquitous taxa were selected from cyanobacterial, cyanobacterial-lichen, and moss-dominated biocrusts, respectively, and also selected from the meta-community. Then, the relationships of α diversity (*H′* and PD) with the relative abundance of ubiquitous taxa were measured.

For phylogenetic analyses, species with a cumulative abundance of 85% in bacterial and eukaryotic communities were retained. The phylogenetic tree was constructed using Fast Tree v2.2.10 with default settings having 500 iterations and 999 bootstraps (Price et al., 2009) and visualized at iTOL.^1^ Meanwhile, the edge-length abundance distribution was calculated from each successional phylogeny (O'Dwyer et al., 2012).

To explore the source of community difference, the Sørensen dissimilarity (β_sor_) in each successional stage was partitioned into the turnover (β_sim_) and nestedness (β_nes_) components (Baselga, 2010). The Sørensen dissimilarity was formulated as follows:


$$\beta sor=\frac{b+c}{2a+b+c}\text{;}$$

Simpson dissimilarity index was used to describe spatial turnover without the influence of richness gradients, formulated as follows:


$$\beta sim=\frac{\min\left(b,c\right)}{a+\min\left(b,c\right)}\text{;}$$

The β_nes_ component was calculated as follows:


βnes=βsor–βsim

In the equations, *a* is the number of species occurring in both paired sites of each successional stage, *b* is the number of species that occur only in one site, and *c* is the number of species occurring in the other site. They were calculated in the package betapart in *R.* βsim/βsor was considered as the turnover ratio (Baselga, 2010), and its correlations with α diversity (Spearman correlation) in the bacterial and eukaryotic community were calculated at local and continental scales, respectively. Environmental data were transformed by the Euclidean distance for the Mantel test (Spearman, permutation = 999, *p < 0.05, **p < 0.01), which was used to investigate the correlations between the turnover ratio and the micro-environmental (including edaphic and biotic factors) and macro-climatic (climatic factors) conditions in each successional stage (the package vegan in *R*) (Oksanen et al., 2015). Then, the highly related factors were selected at local and continental scales through Spearman correlation analysis.

### 2.3. Investigation of the biogeographic pattern

To compare the effects of primary succession and biogeography on community differences, we used the samples from four local scales. Community differences were calculated by analysis of similarities (ANOSIM; permutation = 999, *p < 0.05, **p < 0.01) based on Bray–Curtis and weighted–UniFrac dissimilarities at the species level, respectively.

We investigated community differences among three successional stages, and each successional stage contained samples from the two sample sites (termed as compared among successional stages). Meanwhile, community differences were compared between two sample sites, and each site contained three successional stages (termed as compared between sample sites). In these two ways of comparisons, vast subsets of combinations were obtained when an alterable number of samples between paired comparisons was used instead of whole samples. Here, community differences were computed using 100 randomly combinational comparisons. Finally, the community differences (*R*^2^) with distance were studied by linear regression, and a schematic is shown in Supplementary Figure S2.

### 2.4. Covariant relationships between biodiversity and the environment

Changes in the covariant relationships between α/β diversity and environmental factors with succession were assessed using multiple linear regression. Environmental factors with high multicollinearity were first excluded, and then the optimum combination of environments was selected by the all-subset regression method (the package leaps in *R*) and checked by the Global Statistical Test (the package gvlma in *R*). When the linear models had the same goodness-of-fit (GoF), the one with a lower Akaike information criterion was selected.

Partial least square path models (PLS-PMs) were also applied to demonstrate the correlations of environmental factors with α and β diversity by using the package plspm in *R*. First, climatic, edaphic, and biotic data were transformed into Z-scores and then their Euclidean distance was calculated, respectively. The distance of integrated α diversity indices (including PD, richness, and Pielou evenness) was also calculated. The Bray-Curtis and weighted–UniFrac dissimilarities were used to indicate the taxonomic and phylogenetic facets of β diversity. Dissimilarity data were introduced into initial PLS-PMs based on an *a priori* conceptual hypothesis, and paths with <0.7 loadings were removed. This process cannot be contrary to logic and was stopped until model matching achieved the optimum fit. Finally, we established PLS-PMs for bacterial and eukaryotic communities in cyanobacterial, cyanobacteria–lichen, and moss-dominated biocrusts, respectively. We quantified the relationships among these variables with path coefficients. The GoF index was used to estimate the prediction performance of models.

## 3. Results

### 3.1. Community structure and phylogenetic characteristics

**Figure 1:**
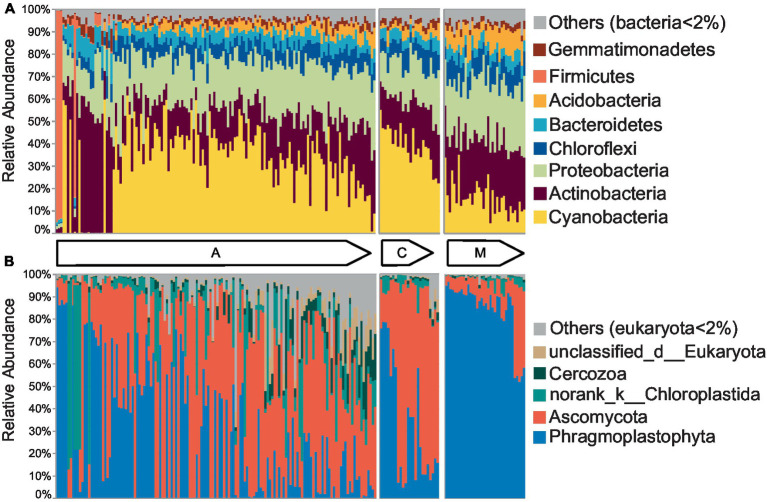
The community structures of biocrusts changed with succession. The community structures of cyanobacterial (A), cyanobacterial-lichen (C), and moss-dominated (M) crusts were separately arranged according to the increased Shannon index in bacteria **(A)** and eukaryotes **(B)** as shown by the arrow direction.

To intuitively display the community structure, 140 cyanobacterial, 24 cyanobacterial-lichen, and 36 moss-dominated biocrust communities were arranged according to ascending *H′*. The bacterial communities were primarily composed of Cyanobacteria with the highest abundance (29.63%), Proteobacteria (22.14%), and Actinobacteria (21.81%) (Figure 1A), whereas the eukaryotic communities were dominated by Ascomycota (39.73%) and Phragmoplastophyta (39.15%) (Figure 1B). The number of phyla having a linear relationship with α diversity at the early successional stage was at least 1.5 times higher than that at the late stage in both communities (Supplementary Table S1), and the multiple was even larger at the class, order, family, and genus levels (Supplementary Table S2). According to the phylogenetic tree (Supplementary Figure S3), the bacterial community had more clades and branching events than eukaryotes. In bacteria and eukaryotes, nearly all branches appeared in the early successional stage, whereas some branches disappeared at the later stage primarily in firmicutes and some Cyanobacteria of bacteria, as well as Phragmoplastophyta of eukaryotes. Thus, the abundance of phyla tended to be stable and that of some branches was excluded from the phylogeny with succession.

### 3.2. The changing pattern of α and β diversity with succession

**Figure 2:**
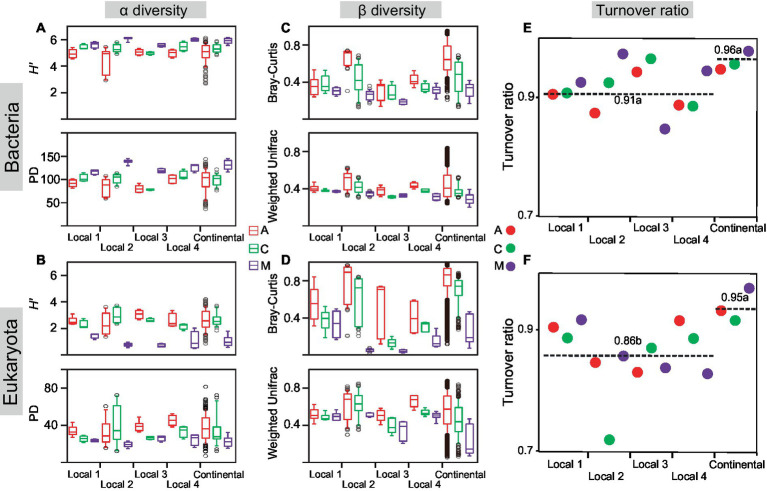
Biodiversity changed with succession. The Shannon index (*H’*) and phylogenetic diversity (PD) were calculated in cyanobacterial (A), cyanobacterial-lichen (C), and moss-dominated (M) crusts at local and continental scales **(A,B)**. Bray-Curtis and Weighted-Unifrac dissimilarities were also calculated **(C,D)**. The turnover ratio of bacteria **(E)** and eukaryotes **(F)** was calculated at different spatial scales **(E,F)**. Significant differences were tested by ANOVA (df = 2, *p* < 0.05) and marked with lowercase letters as mean values decreased.

*H′* and PD were synchronously used to indicate the change feature of α diversity with successional stages (Figures 2A,B; Supplementary Table S3). At the local and continental scales, the α diversity indices in both communities gradually changed with the primary succession in a small range, whereas the α diversity of bacteria increased and that of eukaryotes decreased. In other words, α diversity of the bacterial community in moss-dominated crusts was two times higher than that of cyanobacterial crusts, whereas α diversity of the eukaryotic community in moss-dominated crusts declined by half than cyanobacterial crusts. Furthermore, we found that *H′* and PD had significant differences between cyanobacterial and moss-dominated crusts rather than between cyanobacterial and cyanobacteria-lichen for bacterial and eukaryotic communities (ANOVA, *p < 0.05). Regardless of the spatial scales, bacterial and eukaryotic α diversity increased and decreased with the primary succession of biocrusts, respectively.

With the succession of biocrusts, the phylogenetic and taxonomic β diversity of bacterial and eukaryotic communities both decreased nearly by half and these values became stable at the late stage (moss crusts) compared with the early stage (cyanobacterial crust) at the local and continental scales (Figures 2C,D; Supplementary Table S3). Sørensen dissimilarity was partitioned into turnover and nestedness components (Figures 2E,F), and the turnover component dominated (>85%) in each successional stage at both spatial scales. Comparison of the ratio of turnover among spatial scales (ANOVA, local scales: *n* = 12; continental scale: *n* = 3) revealed no significant difference in bacteria, but the turnover of eukaryotes increased at larger spatial scales. Regardless of spatial scales, consistently positive or negative correlations existed between turnover ratio and α diversity in bacteria and eukaryotes, respectively (Supplementary Table S4). The sources of community differences in each successional stage were dominated by the turnover component, but the correlations of turnover ratio with α diversity were opposite between bacterial and eukaryotic communities.

The correlations of turnover ratio with environments were explored from the perspective of succession (Supplementary Table S5) and spatial scale (Supplementary Figure S4), respectively. For the succession, the turnover ratio was negatively correlated with environmental conditions, and relatively higher with the micro-environment at the early cyanobacterial stage. Conversely, the turnover ratio was positively correlated with environmental factors, and higher with macro-climate at the late moss stage. For the spatial scale, the turnover ratio was correlated with micro-environments, especially with edaphic factors and biotic activities at the local scale. Significant correlations between turnover ratio and macro-climatic factors, specifically windspeed, primarily occurred at the continental scale.

### 3.3. Balanced biogeographic pattern between distance-decay and succession

**Figure 3:**
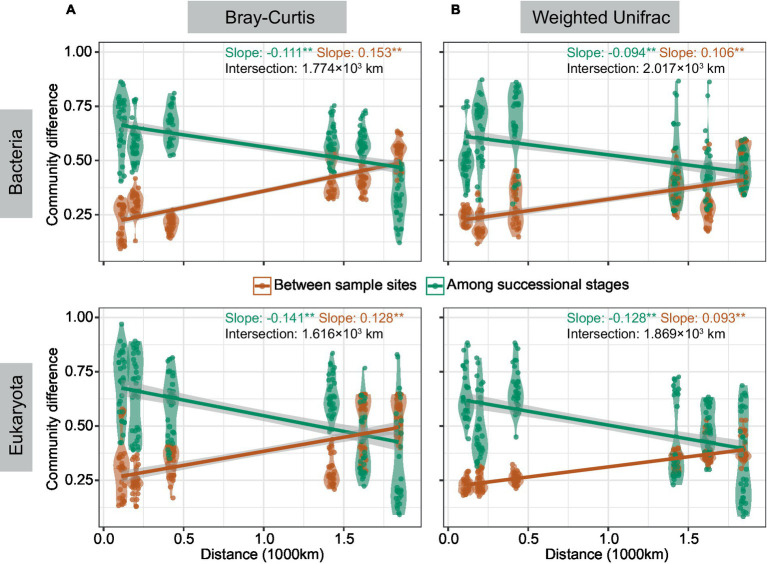
The patterns of community differences changed with distance. Community differences were studied by ANOSIM (*p < 0.05, **p < 0.01) based on Bray-Curtis **(A)** and weighted-UniFrac **(B)** dissimilarity. Linear regression was conducted between community differences and distance. The red line represents differences among successional stages, and the green line represents differences between two sample sites with kilometers of distance. The distance corresponding with the intersection of the two lines was calculated.

Community differences among successional stages and between integral sample sites were calculated, and then the change trend of the two difference values with distance was studied (Figure 3). Results demonstrated that community differences among the three successional stages decreased with distance and that of paired sample sites increased with distance regardless of taxonomic Bray-Curtis or phylogenetic weighted-UniFrac dissimilarity. The distance corresponding with the intersection of two opposite patterns in bacteria (1.77 × 10^3^ and 2.01 × 10^3^ km based on Bray-Curtis and weighted-UniFrac respectively) was larger than that in eukaryotes (1.61 × 10^3^ and 1.86 × 10^3^ km based on Bray-Curtis and weighted-UniFrac respectively). In summary, we found reduced community differences among successional stages with distance, as well as pronounced community differences between sample sites with distance.

### 3.4. Relationships of habitat environments and biodiversity with succession

**Figure 4:**
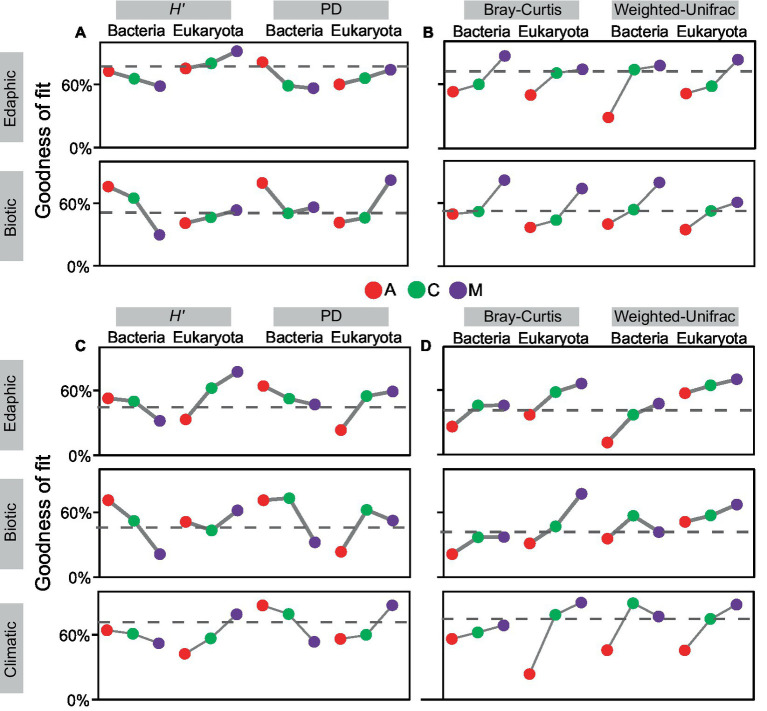
Covariant relationships existed between biodiversity and environmental factors with succession. Environmental factors were divided into three categories: edaphic, climatic, and biotic factors. The goodness-of-fits of α/β diversity and environments were calculated at the local scale **(A,B)** and continental scale **(C,D)** in cyanobacterial (A), cyanobacterial-lichen (C), and moss-dominated (M) crusts. The dashed line in the box indicates the average values.

At local and continental scales (Figure 4), the covariation of α diversity (*H′* and PD) with environments commonly decreased in bacteria and increased in eukaryotes, whereas the covariation of β diversity (Bray–Curtis and weighted-UniFrac) with environments mostly increased in both communities. Comparatively, the GoFs of edaphic factors with biodiversity were generally greater at local scales (α diversity: 0.69 ± 0.11, β diversity: 0.63 ± 0.16) than that at the continental scale (α diversity: 0.51 ± 0.15, β diversity: 0.46 ± 0.17). Besides, the GoFs of climatic factors (α diversity: 0.64 ± 0.14, β diversity: 0.66 ± 0.21) were greater than that of edaphic factors (α diversity: 0.51 ± 0.15, β diversity: 0.46 ± 0.17) at the continental scale. However, the covariation of biotic factors and biodiversity mostly showed no regularity with succession especially manifesting in the phylogenetic facet. In general, environmental constraints on changes in successional α diversity were opposite between bacteria and eukaryotes, whereas its constraints on changes in successional β diversity were alike in both communities. Their biodiversity changes with succession were also dominantly constrained by edaphic and climatic environments at local and continental scales, respectively.

### 3.5. Regulation of biodiversity maintenance in biocrusts

**Figure 5:**
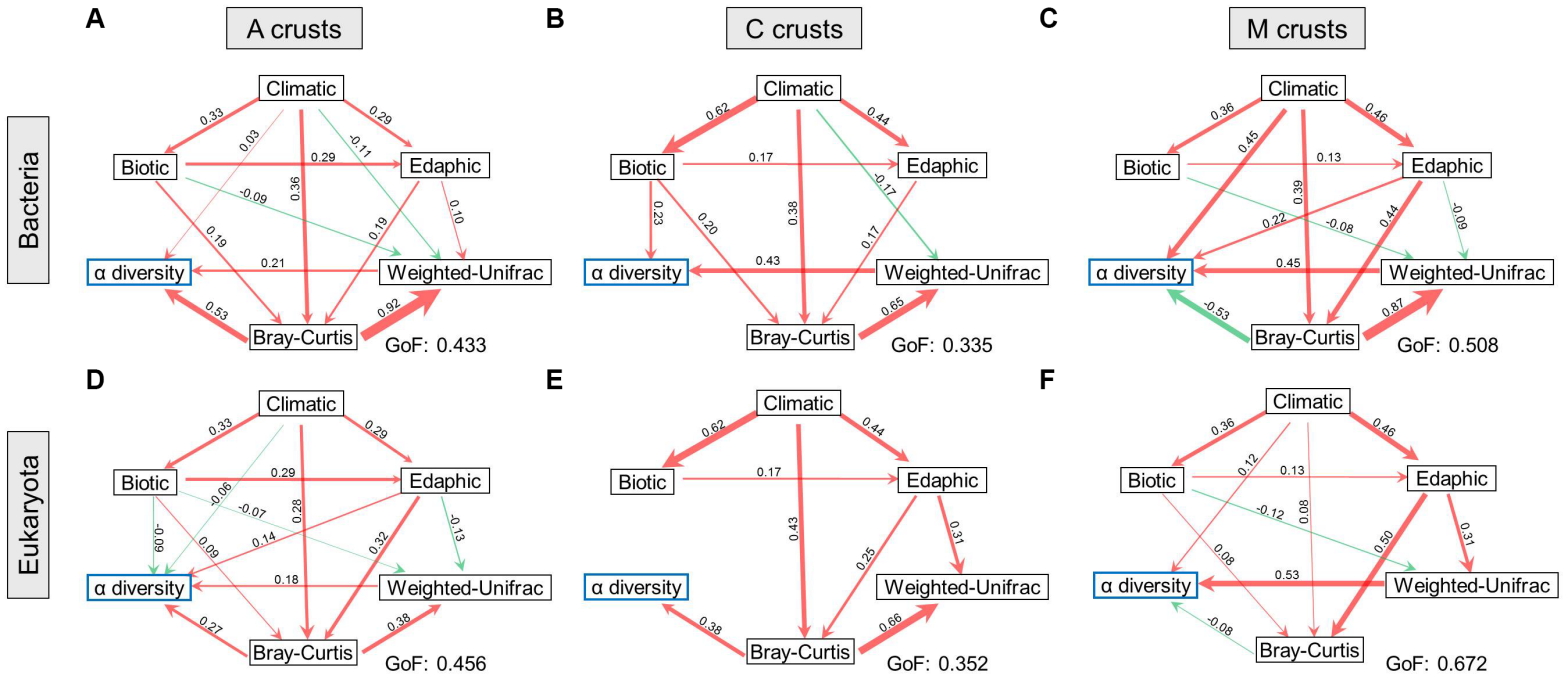
Biodiversity maintenance was regulated in biocrusts. The relationships between biodiversity and environments were demonstrated at cyanobacterial (A), cyanobacterial-lichen (C), and moss-dominated (M) crusts in bacteria **(A–C)** and eukaryotes **(D–F)**. The width of the arrow lines indicates the strength of the relationships (*p* < 0.05). Red and green arrows indicate positive and negative relationships, respectively. The values of goodness-of-fit (GoF) are marked, respectively.

In bacteria, only climatic factors had a positive effect on the integrated α diversity of the early successional stage (Figures 5A,D). In eukaryotes, climatic, edaphic, and biotic factors all exerted influences. Integrated α diversity was also positively affected by β diversity. In terms of β diversity, climatic factors were the most influential environments in bacteria, which had positive and negative effects on the taxonomic and phylogenetic facets, respectively. In eukaryotes, edaphic and biotic factors had positive and negative effects on the taxonomic and phylogenetic facets, respectively.

The integrated α diversity of the middle successional stage (Figures 5B,E) was positively affected by the taxonomic facet of β diversity in both communities. In bacteria, it was additionally affected by biotic factors. In terms of β diversity, taxonomic β was positively influenced by climatic and edaphic factors in bacteria and eukaryotes, and that in bacteria was additionally affected by biotic factors. Conversely, the phylogenetic β was negatively affected by climatic factors in bacteria, and that in eukaryotes was positively affected by edaphic factors.

The integrated α diversity of the late successional stage (Figures 5C,F) was positively affected by climatic factors in both communities. In contrast to the early and middle successional stages, taxonomic β diversity negatively affected integrated α diversity in both communities. In terms of β diversity, taxonomic β was positively affected by climatic and edaphic factors in bacteria and eukaryotes, whereas phylogenetic β diversity was negatively and positively affected by edaphic factors in bacteria and eukaryotes, respectively.

In summary, climatic factors were the most conspicuous environments affecting integrated α diversity. Intriguingly, integrated α diversity tended to be negatively affected by taxonomic β diversity with succession, whereas taxonomic β diversity was consistently regulated positively by environments. Phylogenetic β diversity was the most negatively regulated by the environments.

## 4. Discussion

### 4.1. Bacterial α diversity increased but eukaryotic diversity decreased with the primary succession

Following the sequence of primary succession, α diversity often increases (Fierer et al., 2010; Dornelas et al., 2014; Maier et al., 2018; Ortiz-Alvarez et al., 2018). In biocrusts, our results showed that the α diversity of bacterial communities increased, whereas that of eukaryotic communities decreased (Figures 2A,B). This finding implied that even microorganisms in the same succession process may have different change patterns of α diversity, which depend on different responses to environmental stresses and phylogenetic properties of different life domains (Hawkes and Keitt, 2015). Interestingly, we found that these patterns in bacteria and eukaryotes did not change with spatial scales. The α diversity of both communities also tended to fluctuate within a smaller range at the late than at the early stage (Figures 2A,B), manifesting the processes of habitat differentiation and landscape isolation (Leibold et al., 2019). Accordingly, the proportion of common OTUs tended to be larger with the forward succession, so we defined the abundance of ubiquitous taxa as precise chronosequence characteristics to show more detailed α-diversity changes (Supplementary Figure S5). On the level of integral succession (Supplementary Figure S5A), our result showed that α diversity did not increase indefinitely with the abundance of ubiquitous taxa, which can function as a threshold for determining α diversity. On the level of separated succession (Supplementary Figure S5B), the community contained a higher abundance of ubiquitous taxa at the middle-late stage corresponding with higher and lower α diversity in bacteria and eukaryotes, respectively. This finding indicated that this threshold may affect the changes in α diversity with succession, which may explain why α diversity showed hierarchical changes despite the community’s continuous. A climax community could also exist in biocrusts (Meiners et al., 2015).

### 4.2. β diversities of both bacteria and eukaryotes decreased with succession at the subcontinental scale

β diversity decreased with succession (Figures 2C,D), proving the results of primary succession in many habitats (Purschke et al., 2013; Dornelas et al., 2014; Ortiz-Alvarez et al., 2018). This phenomenon can be considered as biotic homogenization driven by environments (Olden, 2006) and has been observed in many terrestrial assemblages (Rodrigues et al., 2013; Mori et al., 2015; Gossner et al., 2016). This result also can be explained by the successional convergence driven by self-organization in chronosequences (O'Sullivan et al., 2019; Xu et al., 2020). Moreover, the number of phyla that had a covariant relationship with α diversity decreased with succession in both communities (Supplementary Tables S1, S2). These findings indicated a tendency for a stable community structure (Vellend et al., 2017). In contrast to biocrust habitats, salt marshes had strong environmental filters that provided more niche partitions (Dini-Andreote et al., 2014), and resulted in community differences in late succession being greater than in the early stage. These contrasting results suggested biocrusts provided fewer environmental filters than expected despite the drought, like previous descriptions of them as fertile islands in dryland (Weber et al., 2016), contributing to descending community differences with succession.

Fundamentally, the sources of community differences in biocrusts were dominated by the turnover component (Figures 2E,F). Despite turnover being a common way of community assembly, it may result in diverse community structures in the same ecosystem (Soininen et al., 2018; Menegotto et al., 2019). Indeed, our results demonstrated the fact that bacterial communities contained more biodiverse phyla than eukaryotes (Figure 1). Therefore, we speculated that the generalists and specialists participated in bacterial and eukaryotic turnover, respectively. Only then can we interpret the results that the turnover ratio was positively and negatively correlated with α diversity in bacteria and eukaryotes, respectively (Supplementary Table S4). In addition, distinguished from increasing with spatial scales in eukaryotes, the turnover ratio showed no difference between local and continental scales in bacteria which may be attributed to their more versatile ecological strategies and smaller body sizes enabling the plasticity of wide distribution species pool (Farjalla et al., 2012; Wu et al., 2018). Only in the presence of a larger species pool in bacteria than in eukaryotes, can more clades be maintained in a bacterial community even at a high turnover ratio (Wang et al., 2013). Meanwhile, larger species pools can also offer more possibilities of assemblages with multifarious microorganisms facilitating an increase of α diversity (Wang et al., 2013; Picazo et al., 2020). Briefly, under the background of gradually stable community structures in the way of a high turnover ratio, bacteria had a larger species pool than eukaryotes, which supported the view that generalists participated mostly in bacterial turnover.

Considering that more generalists participated in bacterial turnover than eukaryotes, we were motivated to further explore it from a phylogenetic perspective, which usually underlies the generation of many ecological theories (O'Dwyer et al., 2015; Graham et al., 2018). The lagging elbow in edge-length abundance distribution demonstrated (Supplementary Figure S6B) that more branching events were distributed into the tree and existing multiple lineages in the early stage than in the middle-late successional stage. This finding suggested that some clades containing more branches coalesced or were removed with succession (O'Dwyer et al., 2015). Specifically, they were Firmicutes and some Cyanobacteria in bacteria and Phragmoplastophyta in eukaryotes (Supplementary Figure S3). Combined with the fact that bacterial α diversity increased and eukaryotic α diversity decreased with succession, we speculated that more closely related species were assembled in a clade and thus exhibiting the merging of coalescence in the bacterial community, whereas, in eukaryotic phylogeny, more species were removed from the community (O'Dwyer et al., 2015). Eventually, both community structures presented successional convergence through turnover participation by different attributive microorganisms.

Furthermore, since that turnover may be driven by specific habitat differentiation (Wang et al., 2017), environmental filtering, and adaptive niche evolution (Leibold et al., 2019), we investigated environments related to turnover ratio at succession (Supplementary Table S5) and spatial scales (Supplementary Figure S4). Results showed that soil texture and biotic factors were related to turnover ratio at the early successional stage and local scale, suggesting the shaping effects of Cyanobacteria on micro-habitats and their active interaction with heterotrophs (Ratzke et al., 2020). Instead, the role of macroclimate (windspeed) and salinity-related edaphic factors (pH, HCO_3_^−^) were manifested at the late succession and continental scale, illustrating the process of dispersal in driving turnover, which validated the mediation of windspeed in biocrust community assembly (Li and Hu, 2021). Overall, our results elucidated that micro-environments and macroclimate alternately maintained high turnover at different spatial scales.

### 4.3. Balanced biogeographic and local environmental effects determined the patterns of microbial biodiversity at local and subcontinental scale

Given that the sampling transect contained complex information on environmental effects, we also studied the changes in β diversity with succession at different spatial scales. Some opinions indeed indicated that β diversity changes depended on the spatial scale (Martiny et al., 2011). Likewise, the decrease in rangeability of β diversity with succession was greater at the continental than at the local scales (Figures 2C,D). A recent study has also proposed successional convergence in biocrusts (Xu et al., 2020), suggesting that convergent communities at a late stage even with farther geographical sites can have more similarity than expected. The convergence amplified community differences among successional stages, but this effect did not remain constant. One possibility was that the force decreased with geographical distance. The reason may be the changes in species pool size across the climate zone, microorganism dispersal, and the maintenance of the environment on turnover (Bryant et al., 2016; Xu et al., 2020), which gradually concealed the effect of successional convergence on observed community differences under the high windspeed in dryland. In other words, the biocrust biogeographic pattern was balanced by distance decay and successional convergence. Furthermore, the intersectional distance of two opposite forces at the phylogenetic facet was greater than that at the taxonomic facet, and that in bacteria was larger than that in eukaryotes (Figure 3). This finding suggested a separated range of species pool in dryland, and that the differentiated species pool of bacteria had a wider spatial range than eukaryotes (Wu et al., 2018). Therefore, the size of the sampling transect-related species pool size determined the observation of biogeographic patterns.

Recent advances have shown the versatile effects on biodiversity from different categories of environments (Garcia-Pichel et al., 2013; Gotelli et al., 2017; Rillig et al., 2019), but whether the consistency of environmental effects on different facets of biodiversity remains unclear. Accordingly, we investigated the correlations of edaphic, climatic, and biotic factors with biodiversity at the taxonomic and phylogenetic facets in biocrusts to improve our understanding of environmental regulation. We found that the relationships of edaphic and climatic factors with bacterial α diversity weakened with succession, but grew strongly in eukaryotes (Figure 4A). Thus, the distinct direction of environmental force between the two communities may contribute to different change patterns of α diversity (Figures 2A,B). The covariant relationship of edaphic and climatic factors with Bray-Curtis increased with succession in bacteria and eukaryotes (Figure 4B), suggesting more distinguished environmental filtering may result in β diversity decline with succession in both communities (Figures 2C,D; Whittaker and Rynearson, 2017). These results implied that the change patterns of taxonomic biodiversity were likely governed by deterministic ecological processes (i.e., edaphic and climatic factors) (Purschke et al., 2013). In this way, in terms of the phylogenetic facets including PD and Weighted-Unifrac dissimilarity, no corresponding relationships existed between biodiversity change patterns and their environmental covariant trends with succession. These discordances suggested that phylogenetic α diversity may be explained by unquantified factors rather than edaphic and climatic ones (Purschke et al., 2013; Le Bagousse-Pinguet et al., 2019). Given that regulations of biotic factors for PD were the strongest in cyanobacterial-lichen crusts, we speculated that complex Cyanobacteria-Ascomycota interactions in symbionts may be unquantified and poorly understood parts of phylogenetic α diversity (Grube et al., 2015). In general, edaphic and climatic factors can provide more useful insights into taxonomic-biodiversity changes, whereas biotic factors probably underlie the pattern of phylogenetic biodiversity.

In dryland, microbial diversity is affected by wind and aridity directly (Bowker et al., 2010), as well as by edaphic and biotic factors (Hu and Liu, 2003; Tighe et al., 2012; Chen et al., 2020), which widely control the abundance of photoautotrophic organisms (Maier et al., 2018). Accordingly, climatic, edaphic, and biotic factors were introduced to study their effects on different aspects of biodiversity-maintenance mechanisms in biocrusts (Figure 5). Previous studies have shown that climates can affect cyanobacterial population size and community structure (Fernandes et al., 2018). This view was further verified by our results that climatic and edaphic factors are continuously dedicated to taxonomic β diversity with succession in both communities (Figure 5). Combined with the fact that bacteria had more biodiverse taxa than eukaryotes, our results suggested climates probably played a vital role in assembling microorganisms throughout biocrust successional stages, corresponding with the finding of windspeed-mediated community assembly pattern in our previous study (Li and Hu, 2021). Notably, biotic factors in biocrusts primarily included photosynthetic pigments and EPS that had non-negligible impacts on shaping microhabitat gradients by providing organic carbon resources for heterotrophs (Colica et al., 2015) and facilitating sand consolidation (Lan et al., 2014). In particular, symbionts in cyanobacterial-lichen crusts can provide more niches for species colonization (Maier et al., 2014), which can be demonstrated by biotic factors promoting bacterial α diversity (Figure 5B). Furthermore, from the perspective of β diversity, environments mostly inhibited phylogenetic β diversity, as crucially embodied in biotic factors. One possible explanation was that biotic factors may affect the ways microbial interactions and further change community functions. Microorganisms with similar ecological adaptability are frequently retained (Leibold et al., 2019), leading to specific assemblages under the context of drought in this process (Neilson et al., 2017). Conversely, edaphic and climatic factors mostly facilitated the taxonomic β diversity, suggesting that assemblage compositions and distribution patterns had significant responses to environmental gradients. In summary, climatic and edaphic factors mostly facilitated α and taxonomic β diversity, and biotic factors mostly inhibited phylogenetic β diversity.

In conclusion, this study demonstrated the biodiversity of bacterial and eukaryotic communities in biocrusts at multispatial scales. First, the sources of biodiversity in biocrusts were dominated by turnover, primarily with the participation of generalists in bacteria and by specialists in eukaryotes. This factor fundamentally contributed to α diversity changes with succession. Microenvironments and macroclimates were also alternately related to high turnover at different successional stages and spatial scales. Second, the α diversity of bacteria increased even in convergent succession, whereas the impact of distance decay on β diversity gradually exceeded successional convergence at a large spatial scale. The two opposite drivers balanced the biogeography of biocrusts and emphasized the importance of sampling transect-related species pool size. Third, environmental constraints affected successional α and β diversity changes, which were more influenced by edaphic factors at the local scale, and by climatic factors at continental scales, respectively. Moreover, edaphic and climatic factors can be focused on studying taxonomic biodiversity, whereas biotic factors can provide more useful insights into phylogenetic biodiversity. Overall, our study provided important insights into understanding the change patterns of biodiversity at multispatial scales.

## Data availability statement

The datasets presented in this study can be found in online repositories. All sequencing reads generated in this study are publicly available through the SRA database under accession number PRJNA640847. The environmental dataset (DOI: 10.6084/m9.figshare.13172411.v1) and the OTU tables based on the bacterial 16S and eukaryotic 18S rDNA amplicon sequencing (DOI: 10.6084/m9.figshare.24523276) can be found in FigShare.

## Author contributions

YL: Data curation, Formal analysis, Funding acquisition, Investigation, Resources, Writing – original draft. FW: Writing – review & editing. HY: Data curation, Investigation, Writing – review & editing. HL: Funding acquisition, Methodology, Supervision, Writing – review & editing. CH: Conceptualization, Funding acquisition, Methodology, Supervision, Project administration, Writing – review & editing.

## Footnotes

^1^ https://itol.embl.de/
