# Corrigendum: Balanced biogeographic and local environmental effects determine the patterns of microbial diversity in biocrusts at multi-scales

**DOI:** 10.3389/fmicb.2024.1374406

**Published:** 2024-02-01

**Authors:** Yuanlong Li, Fengdi Wang, Haijian Yang, Hua Li, Chunxiang Hu

**Affiliations:** ^1^Hunan Provincial Key Laboratory of Carbon Neutrality and Intelligent Energy, School of Resource and Environment, Hunan University of Technology and Business, Changsha, China; ^2^Key Laboratory of Algal Biology, Institute of Hydrobiology, Chinese Academy of Sciences, Wuhan, China; ^3^Institute of Hematology, Union Hospital, Tongji Medical College, Huazhong University of Science and Technology, Wuhan, China

**Keywords:** biogeography, biological soil crusts, cyanobacteria, microbial diversity, multiple spatial scales, primary succession, species turnover

In the published article, there were two spelling mistakes in the abstract and introduction. The term MiSeq was displayed as “MeSeq”. The correct statement is “MiSeq”.

A correction has been made to the method section of the Abstract. The sentence previously stated:

“(Based on the Illumina MeSeq 16S/18S rRNA sequencing technology, we…)”

The corrected sentence appears below:

“(Based on the Illumina MiSeq 16S/18S rRNA sequencing technology, we…)”

A correction has been made to paragraph 4 of the Introduction. The sentence previously stated:

“(Illumina MeSeq 16S/18S rRNA sequencing was used to examine the…)”

The corrected sentence appears below:

“(Illumina MiSeq 16S/18S rRNA sequencing was used to examine the…)”

The authors apologize for this error and state that this does not change the scientific conclusions of the article in any way. The original article has been updated.

