# Supplementary material for: Balanced biogeographic and local environmental effects determine the patterns of microbial diversity in biocrusts at multi-scales
Source: Front Microbiol. 2023 Nov 9;14:1284864. doi: 10.3389/fmicb.2023.1284864 (PMC10666793; doi:10.3389/fmicb.2023.1284864)
Supplement: Supplementary file 1 [file Data_Sheet_1.docx]

**ORIGNAL ARTICLE**

**Balanced biogeographic and local environmental effects determine the patterns of microbial diversity in biocrusts at multi-scales**

**Running title:** **Microbial diversity of biocrusts at multi-scales**

Yuanlong Li ^a,b^, Fengdi Wang ^c^, Haijian Yang ^b^, Hua Li ^b,*^, Chunxiang Hu ^b^

*^a^ Hunan Provincial Key Laboratory of Carbon Neutrality and Intelligent Energy, School of Resource & Environment, Hunan University of Technology and Business, Changsha 410000, China.*

*^b^ Key Laboratory of Algal Biology, Institute of Hydrobiology, Chinese Academy of Sciences, Wuhan 430072, China.*

*^c^ Institute of Hematology, Union Hospital, Tongji Medical College, Huazhong University of Science and Technology, Wuhan 430022, China.*

*Corresponding author: Hua Li. E-mail: lih@ihb.ac.cn

**This file includes:**

**Supplementary Figures 1 to 6**

**Supplementary Tables 1 to 5**

**Supplementary Methods**

**Supplementary References**

**Figures**


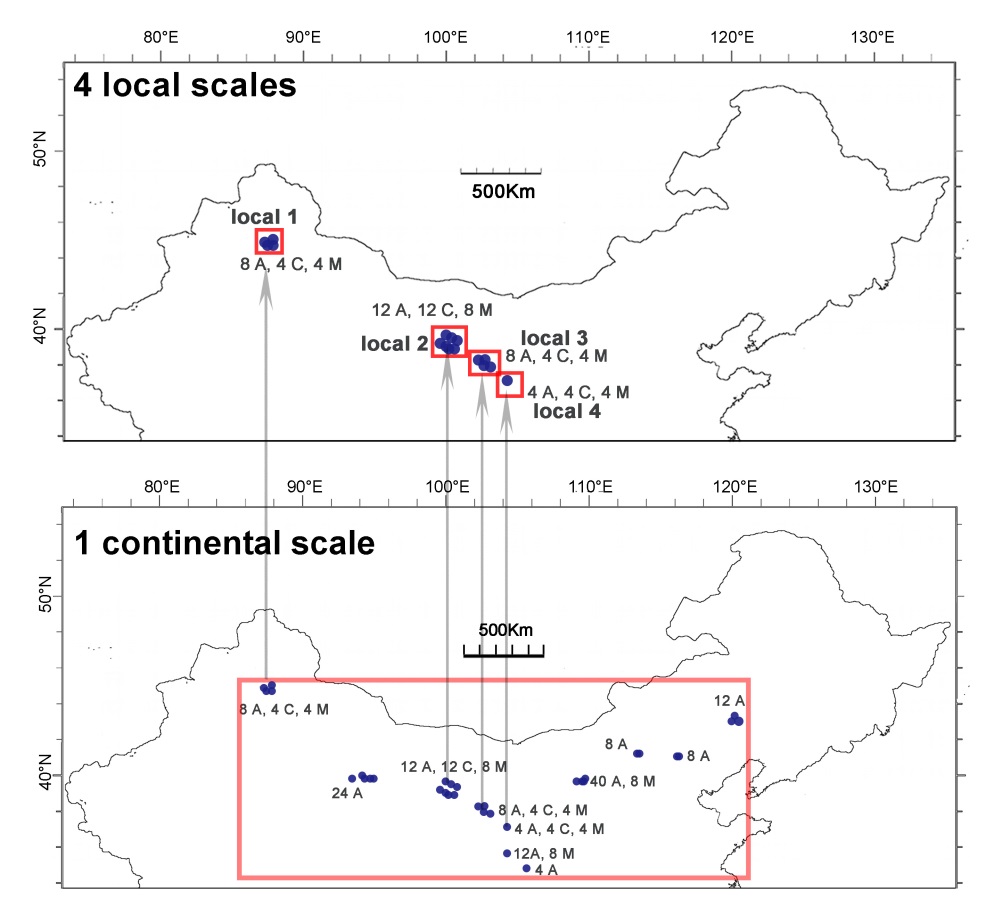


**Supplementary Figure 1. Spatial scales and sampling sites of north China.** The dark blue dots represent the sampling sites, with numbers and letters next to them indicating the number of samples and successional stages, respectively (cyanobacterial-algae (A), cyanobacterial-lichen (C), and moss (M) biocrusts). We obtained four local and one continental scales as shown by red box.


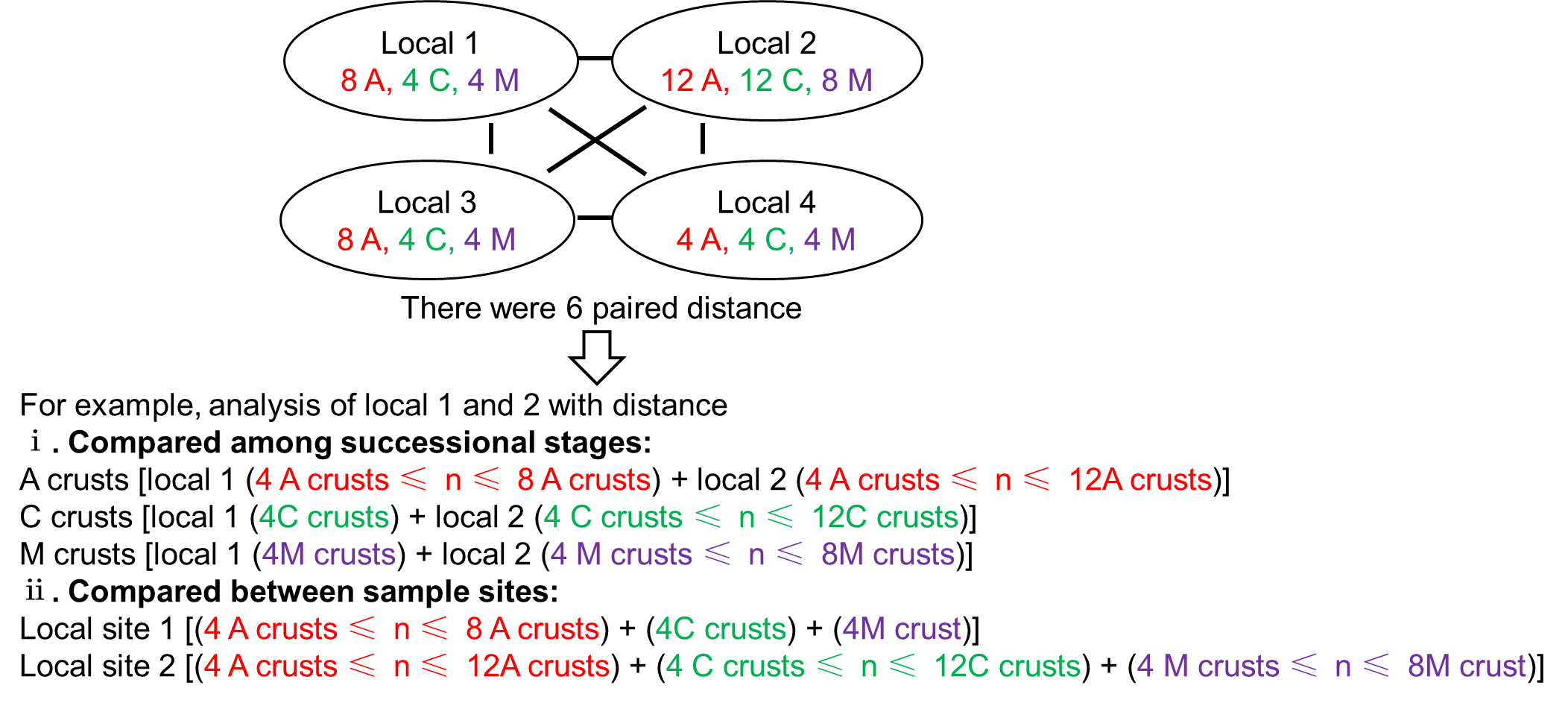


**Supplementary Figure 2. Schematic comparing community differences in biogeographic pattern.** The regional scale had six combinations of pairs from four local sites. Local sites 1 and 2 are taken as examples. Community differences were compared among the three successional stages, and each successional stage contained samples from local sites 1 and 2 (termed as compared among successional stages). Meanwhile, community differences were compared between local sites 1 and 2. Each site contained three successional stages (termed as compared between sample sites). These two ways of comparisons yielded vast subsets of combinations when containing alterable number of samples (at least four) between paired distance instead of whole samples. Here, community differences were computed for 100 randomly combinations. Finally, these value of ANOSIM (R^2^) with distance were studied by linear regression.


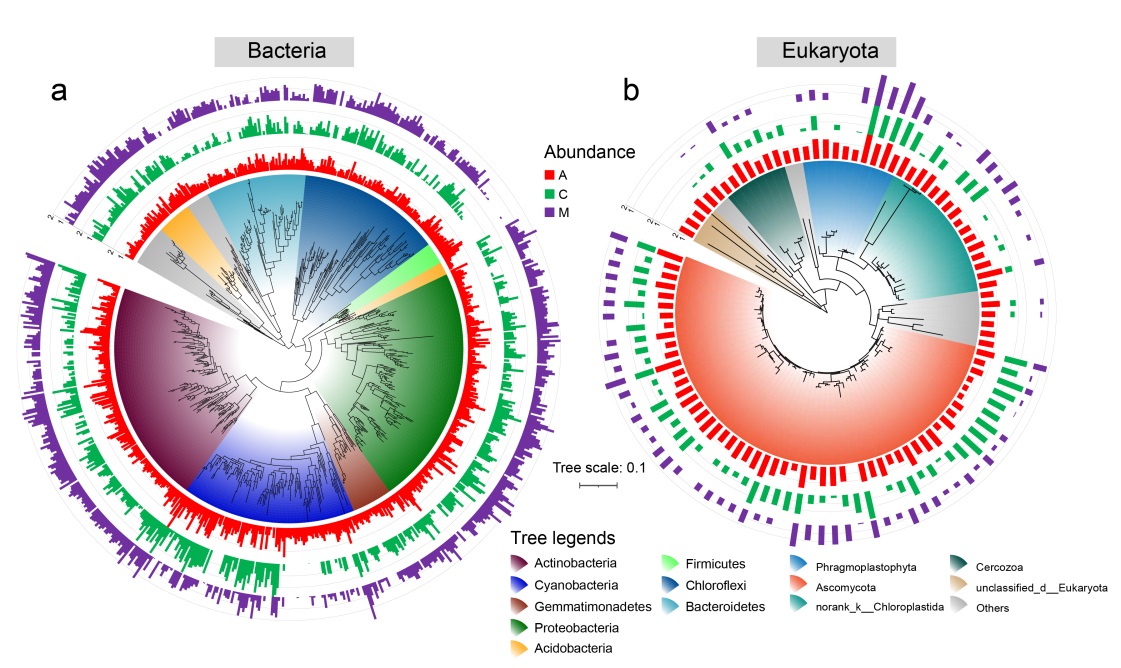


**Supplementary Figure 3. Phylogram of bacterial and eukaryotic communities.** The surrounding bar charts are the average abundance (lg10) of OTUs in cyanobacterial-algae (A), cyanobacterial-lichen (C), and moss (M) crusts.

**
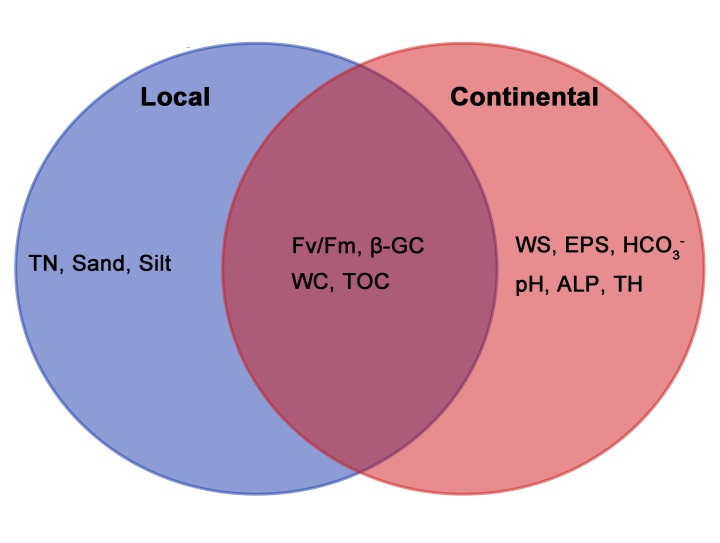
**

**Supplementary Figure 4.** **Venn diagram of environmental factors related to the turnover ratio at different spatial scales.** Spearman correlation was performed between each environmental factor with turnover ratio (βsim/βsor). Environmental factors were transformed into Z-scores. We screened out environmental factors with significant correlations (*p* < 0.05) at different spatial scales and then demonstrated by Venn diagram. Abbreviation: soil-β-glucosidase (β-GC), variable fluorescence/maximal fluorescence (Fv/Fm), scytonemin (Scyt.), chlorophyll *a* (Chl *a*), gross photosynthesis/respiration (Pg/R), total organic carbon (TOC), water content (WC), thickness (TH.), soil alkaline phosphatase (ALP), total nitrogen (TN), extracellular polysaccharide (EPS), altitude (Alt.), and windspeed (WS).


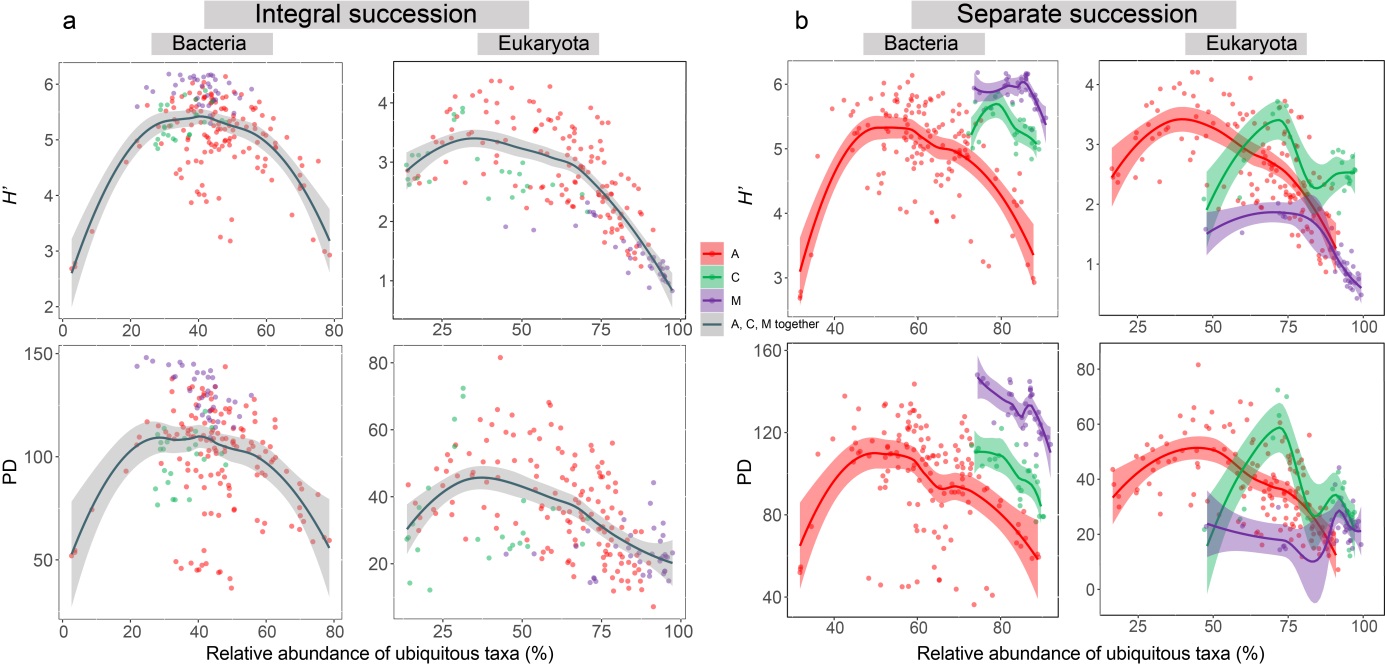


**Supplementary Figure 5. Trajectories of α diversity with relative abundance of ubiquitous taxa.** α diversity included Shannon index (*H'*) and phylogenetic diversity (PD). We defined the OTUs which occurred in >85% of samples as ubiquitous taxa. The widespread taxa were selected from all biocrust samples, referred to as integral succession (a). Likewise, the ubiquitous taxa were selected from cyanobacterial-algae (A), cyanobacterial-lichen (C), and moss-dominated (M) crusts respectively, referred to as separate succession (b). The scattered dots were fitted by a smooth curve, and the shadow was the 95% confidence interval.


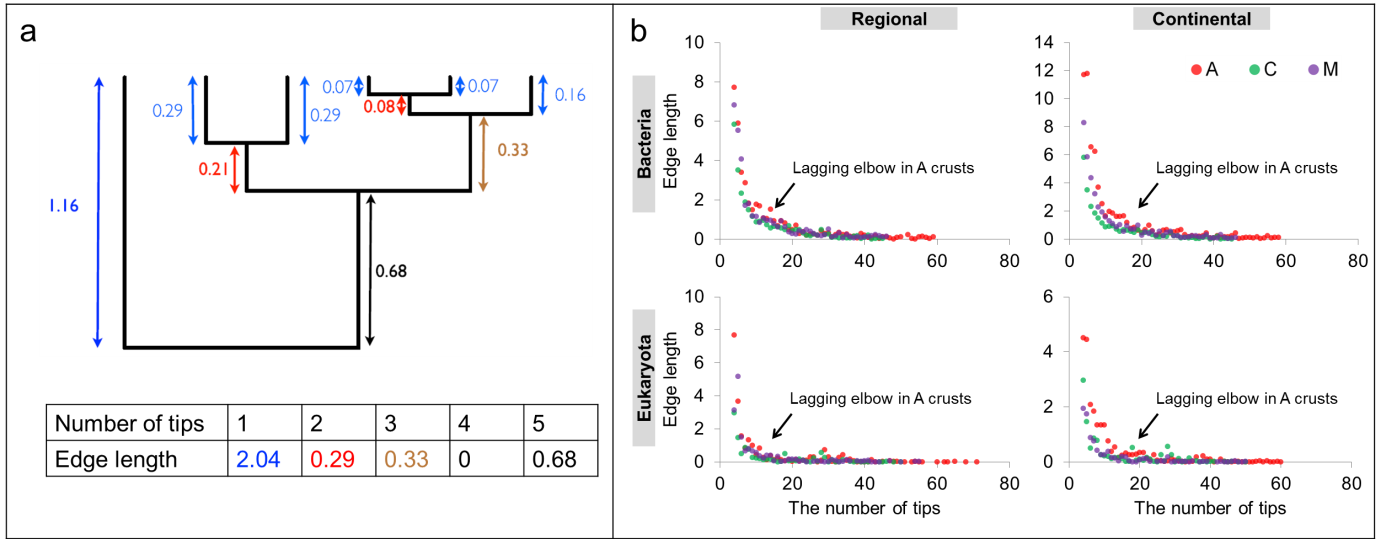


**Supplementary Figure 6.** **Phylogenetic characteristics of different succession stages.** The edge-length abundance distribution (EAD) was defined by focusing on all clades with a given number of descendant tips. Different number of tips can belong to a clade, and each clade had corresponding edge lengths that were the sum of the edge lengths between the node defining each clade and its immediate ancestor. For example, (a) (refers to O’Dwyer’s study (O'Dwyer et al., 2015)), there were clades with 1, 2, 3, and 5 descendant tips in this simulated tree, corresponding 2.04, 0.29, 0.33, 0, 0.68 edge length. (b) To make a detailed comparison of EADs between successional stages in biocrusts, only the elbow fraction was plotted. Phylogenies were computed using OTUs included in bacterial and eukaryotic communities at the regional and continental scales, respectively. Different dot colors indicated cyanobacterial-algae crust (A), cyanobacterial-lichen (C), and moss-dominated (M) crusts.

.

**Tables**

**Supplementary Table 1**. **Linear slope of phylum taxa abundance with α diversity.** Linear regression was used to find phyla whose abundance changed most dramatically (i.e., slope) with *H'* and phylogenetic diversity (PD) in bacteria and eukaryota. The bold font indicated significant change (**p* < 0.05, ***p* < 0.01).

|  |  |  | *H'* |  |  |  | PD |  |
| --- | --- | --- | --- | --- | --- | --- | --- | --- |
|  |  | A-Slope (Ave.) | C-Slope (Ave.) | M-Slope (Ave.) |  | A-Slope (Ave.) | C-Slope (Ave.) | M-Slope (Ave.) |
| **Bacteria** | Cyanobacteria | **0.054**** | **-0.237**** | **-0.176**** |  | **0.003**** | **-0.004**** | -0.002 |
|  | Actinobacteria | **-0.036*** | **0.075**** | 0.04 |  | **-0.002**** | 0.001 | -0.001 |
|  | Proteobacteria | **0.046**** | **0.073*** | **0.077**** |  | **0.001**** | 0.001 | 0.001 |
|  | Chloroflexi | **0.019**** | **0.042*** | 0.004 |  | **0.000**** | 0.001 | **0.001*** |
|  | Bacteroidetes | **-0.006*** | 0.007 | 0.011 |  | **0.000**** | 0.000 | 0.000 |
|  | Acidobacteria | **0.020**** | **0.027*** | 0.031 |  | **0.001**** | **0.001**** | 0.000 |
|  | Gemmatimonadetes | -0.001 | 0.006 | 0.005 |  | **0.000**** | 0.000 | 0.000 |
|  | Firmicutes | **-0.102**** | 0.000 | 0.001 |  | **-0.002**** | 0.000 | 0.000 |
|  | Others (<2%) | **0.006**** | 0.006 | 0.008 |  | 0.000 | 0.000 | 0.000 |
|  |  |  |  |  |  |  |  |  |
| **Eukaryota** | Ascomycota | **0.062*** | **0.233*** | **0.211**** |  | -0.002 | 0.002 | -0.005 |
|  | Phragmoplastophyta | **-0.223**** | **-0.33**** | **-0.244**** |  | **-0.006**** | -0.004 | 0.005 |
|  | norank_k__Chloroplastida | -0.003 | -0.018 | **0.028**** |  | 0.000 | **-0.001*** | -0.001 |
|  | Cercozoa | **0.049**** | **0.018**** | 0.000 |  | **0.002**** | **0.001**** | **0.000**** |
|  | unclassified_d__Eukaryota | **0.036**** | **0.029**** | 0.000 |  | **0.002**** | **0.001**** | **0.000**** |
|  | Others (<2%) | **0.079**** | **0.069**** | 0.005 |  | **0.004**** | **0.002**** | **0.000*** |

**Supplementary Table 2.** **Number of phyla whose abundance had covariance with α diversity at different taxonomic levels.** Linear regression was used to detect the number of taxa whose abundance changed significantly (*p* < 0.05) with Shannon index (*H'*) and phylogenetic diversity (PD) at different taxonomic levels in cyanobacterial-algae (A), cyanobacterial-lichen (C), and moss-dominated (M) crusts.

|  | Bacteria | | | | | |  | Eukaryota | | | | | |
| --- | --- | --- | --- | --- | --- | --- | --- | --- | --- | --- | --- | --- | --- |
|  |  | *H'* |  |  | PD |  |  |  | *H'* |  |  | PD |  |
|  | A | C | M | A | C | M |  | A | C | M | A | C | M |
| Phylum | 8 | 5 | 2 | 8 | 2 | 1 |  | 5 | 5 | 3 | 4 | 4 | 3 |
| Class | 25 | 12 | 6 | 27 | 11 | 12 |  | 30 | 31 | 11 | 30 | 33 | 20 |
| Order | 50 | 17 | 11 | 52 | 22 | 33 |  | 37 | 42 | 16 | 36 | 42 | 21 |
| Family | 75 | 26 | 14 | 74 | 35 | 51 |  | 41 | 51 | 19 | 41 | 47 | 23 |
| Genus | 108 | 40 | 21 | 109 | 57 | 73 |  | 53 | 62 | 24 | 52 | 58 | 27 |

**Supplementary Table 3**. **Detailed statistical results of biodiversity changes with succession.** Values in the table correspond with Fig. 1 in the main text and presented as the mean±SD. ANOVA was used to compare significant differences of α and β diversity indices among succession stages. The significances (least Significant difference, *p* < 0.05) were marked by lowercase. Different lowercases **a**, **b**, and **c** represent significant differences as values decreased.

|  |  | **α diversity** | | | | | | | | | | | | |
| --- | --- | --- | --- | --- | --- | --- | --- | --- | --- | --- | --- | --- | --- | --- |
|  |  | **Bacteria** | | | | | |  | | **Eukaryota** | | | | |
|  |  | Df | F | | **A** **c**rust | **C** **c**rust | M **c**rust |  | Df | | F | **A** **c**rust | **C** **c**rust | M **c**rust |
| **Local 1** | *H’* | 2 | 10.242 | | 4.92±0.31**c** | 5.42±0.18**b** | 5.62±0.24**a** |  | 2 | | 15.518 | 2.58±0.27**a** | 2.27±0.31**b** | 1.47±0.2**c** |
| **Local 2** | *H’* | 2 | 17.936 | | 4.83±0.95**c** | 5.35±0.3**b** | 6.09±0.12**a** |  | 2 | | 16.119 | 2.36±0.78**a** | 2.95±0.53**a** | 0.78±0.13**b** |
| **Local 3** | *H’* | 2 | 14.304 | | 5.06±0.21**b** | 4.99±0.11**b** | 5.56±0.12**a** |  | 2 | | 18.469 | 3.07±0.32**a** | 2.62±0.12**b** | 0.71±0.12**c** |
| **Local 4** | *H’* | 2 | 15.758 | | 4.98±0.28**c** | 5.47±0.33**b** | 5.99±0.1**a** |  | 2 | | 15.422 | 2.56±0.54**a** | 2.18±0.23**a** | 1.07±0.67**b** |
| **Continental** | *H’* | 2 | 33.175 | | 4.97±0.75**c** | 5.32±0.3**b** | 5.89±0.23**a** |  | 2 | | 32.413 | 2.64±0.79**a** | 2.66±0.52**a** | 1.07±0.43**b** |
|  |  |  |  | |  |  |  |  |  | |  |  |  |  |
| **Local 1** | PD | 2 | 19.718 | | 92.06±7.37**c** | 102.62±8**b** | 116.49±5.02**a** |  | 2 | | 14.633 | 33.9±5.22**a** | 25.57±3.04**b** | 23.34±1.26**b** |
| **Local 2** | PD | 2 | 12.422 | | 83.39±17.88**c** | 102.01±10.1**b** | 139.06±4.35**a** |  | 2 | | 25.295 | 31.9±13.31**a** | 39.79±20.87**a** | 20.02±3.31**b** |
| **Local 3** | PD | 2 | 11.72 | | 79.87±8.06**b** | 78.54±1.28**b** | 118.72±4.03**a** |  | 2 | | 14.551 | 39.04±5.32**a** | 26.47±1.53**b** | 28.85±3.69**b** |
| **Local 4** | PD | 2 | 25.589 | | 101.15±9.2**c** | 108.23±9.37**b** | 126.71±8.43**a** |  | 2 | | 21.512 | 45.33±5.84**a** | 33.48±5.95**b** | 24.81±6.08**c** |
| **Continental** | PD | 2 | 33.465 | | 97.36±25.1**b** | 99.23±12.78**b** | 131.32±10.85**a** |  | 2 | | 34.352 | 37.56±14.48**a** | 28.15±15.94**b** | 23.19±6.74**c** |
|  |  | **β diversity** | | | | | | | | | | | | |
|  |  | **Bacteria** | | | | | |  | | **Eukaryota** | | | | |
|  |  | Df | F | **A** **c**rust | | **C** **c**rust | M **c**rust |  | | Df | F | **A** **c**rust | **C** **c**rust | M **c**rust |
| **Local 1** | Bray-Curtis | 2 | 23.36 | 0.36±0.11**a** | | 0.38±0.09**a** | 0.3±0.04**b** |  | | 2 | 17.101 | 0.55±0.2**a** | 0.38±0.11**b** | 0.33±0.13**c** |
| **Local 2** | Bray-Curtis | 2 | 14.96 | 0.69±0.23**a** | | 0.54±0.17**b** | 0.26±0.05**c** |  | | 2 | 14.429 | 0.61±0.34**a** | 0.5±0.29**b** | 0.05±0.02**c** |
| **Local 3** | Bray-Curtis | 2 | 17.404 | 0.3±0.11**a** | | 0.29±0.08**a** | 0.19±0.03**b** |  | | 2 | 24.895 | 0.49±0.29**a** | 0.13±0.05**b** | 0.04±0.02**c** |
| **Local 4** | Bray-Curtis | 2 | 16.249 | 0.42±0.06**a** | | 0.34±0.05**b** | 0.31±0.06**c** |  | | 2 | 26.753 | 0.4±0.17**a** | 0.31±0.05**b** | 0.15±0.08**c** |
| **Continental** | Bray-Curtis | 2 | 419.54 | 0.66±0.17**a** | | 0.49±0.08**b** | 0.35±0.06**c** |  | | 2 | 359.06 | 0.81±0.16**a** | 0.69±0.16**b** | 0.27±0.16**c** |
|  |  |  |  |  | |  |  |  | |  |  |  |  |  |
| **Local 1** | Weighted Unifrac | 2 | 19.241 | 0.4±0.04**a** | | 0.38±0.01**b** | 0.37±0.01**b** |  | | 2 | 22.925 | 0.52±0.07**a** | 0.49±0.04**b** | 0.5±0.04**b** |
| **Local 2** | Weighted Unifrac | 2 | 20.303 | 0.5±0.1**a** | | 0.42±0.07**b** | 0.38±0.02**c** |  | | 2 | 24.284 | 0.62±0.15**a** | 0.62±0.14**a** | 0.59±0.07**b** |
| **Local 3** | Weighted Unifrac | 2 | 12.997 | 0.41±0.04**a** | | 0.31±0.01**b** | 0.33±0.01**b** |  | | 2 | 16.041 | 0.5±0.07**a** | 0.39±0.08**b** | 0.35±0.1**c** |
| **Local 4** | Weighted Unifrac | 2 | 20.729 | 0.43±0.03**a** | | 0.38±0.02**b** | 0.31±0.04**c** |  | | 2 | 20.677 | 0.66±0.06**a** | 0.54±0.03**b** | 0.5±0.04**c** |
| **Continental** | Weighted Unifrac | 2 | 308.06 | 0.44±0.45**a** | | 0.38±0.21**b** | 0.31±0.11**c** |  | | 2 | 439.12 | 0.56±0.14**a** | 0.42±0.12**b** | 0.18±0.14**c** |

**Supplementary Table 4**. **Correlations between turnover ratio and α diversity in succession.** β diversity was partitioned using Baselga's method. βsim/βsor was considered as turnover ratio. The α diversity indices including Shannon (*H'*), Pielou evenness, richness, and phylogenetic diversity (PD) were used to calculate the correlation (Spearman, two-tailed, **p* < 0.05, ** *p* < 0.01) with turnover ratio. A gradient from red to blue shading indicates a value from high to low.

|  | **Local scale** | | | |  | **Continental scale** | | | |
| --- | --- | --- | --- | --- | --- | --- | --- | --- | --- |
|  | *H'* | Pielou | Richness | PD |  | *H'* | Pielou | Richness | PD |
| Bacterial turnover ratio | 0.418** | 0.529** | 0.273* | 0.244* |  | 0.532** | 0.519** | 0.473** | 0.449** |
| Eukaryotic turnover ratio | -0.093 | -0.101 | -0.236* | -0.284* |  | -0.276** | -0.229** | -0.522** | -0.532** |

**Supplementary Table 5**. **Correlation between turnover ratio and environments with succession.** Turnover ratio (i.e., βsim/βsor) was calculated based on all biocrust samples during succession. The environmental factors were divided into two categories: microenvironments and macroclimates. They were subjected to Euclidean transformation. Mantel test (Spearman, permutation =999, **p* < 0.05, ***p* < 0.01) was performed with turnover ratio and environmental factors. A gradient from red to blue shading indicates a value from high to low.

|  | **Bacteria (turnover ration)** | | |  | **Eukaryota (turnover ration)** | | |
| --- | --- | --- | --- | --- | --- | --- | --- |
|  | A | C | M |  | A | C | M |
| Macroclimates | -0.127** | 0.045 | 0.219** |  | 0.121 | 0.035 | 0.312** |
| Microenvironments | -0.116** | 0.091 | 0.125* |  | -0.187** | -0.276** | 0.189** |

**Supplementary Methods**

**The classification of biocrusts successional stages**

In order to ensure the consistency and standard of same successional biocrusts, sampling criteria were that cyanobacterial-algae crusts had a flat, light and close to the bare sand color surface, with coverage of nearly 90% organisms cyanobacteria. Cyanobacterial-lichen crusts had a deep black color and colloidal thallus. The symbionts were cyanobacteria and fungi, with surface coverage of about 95%. Moss-dominated crusts were light green and had a blanket shape, covered by *Bryum argenteum* (or *Didymodon nigrescen* and *Tortula bidentata*), with surface coverage of about 95%. The classifications of biocrusts successional stages were referred to previous studies (Lan et al., 2012), and as shown below.


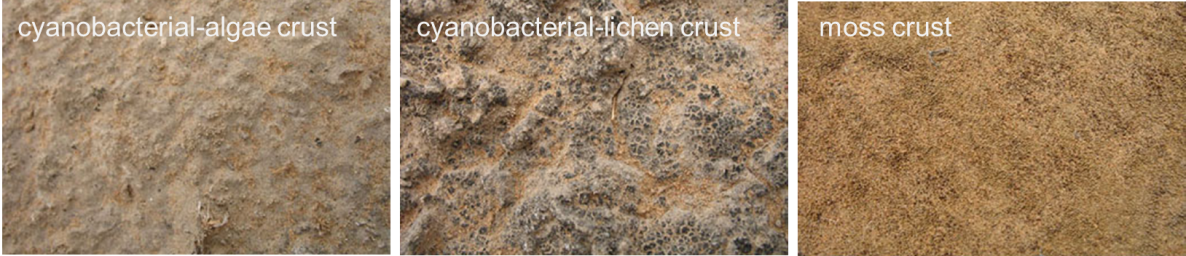


**The determinations of microenvironmental variables**

Soil texture (%) was determined by gravity settling method. Water content (WC, %) was determined by oven-drying method. For pH determinations, each ground and weighed sample was mixed with distilled water at a ratio of 1:5. After mixing and centrifuging thoroughly, part of the supernatant was taken for pH measurements with a YSI-pH100 meter (Yellow Springs, Ohio, USA). Then, the other part of the supernatant was filtered and analyzed for ion content (μmol∙g^−1^) by ion chromatography (Thermo Scientific™ Dionex™ ICS-5000+, Thermo Fisher Scientific, USA) with a C18 column (CNW IC Guard C18 column, CNW Technologies GmbH, Düsseldorf, Germany). The cation and anion concentrations were respectively calibrated using Dionex Six Cation Standard II (K^+^, Ca^2+^, Na^+^, Mg^2+^, and NH_4_^+^) and Dionex Seven Anion Standard II (Cl^−^, SO_4_^2−^, NO_3_^−^, NO_2_^−^, and PO_4_^3−^). The measurement of HCO_3_^−^ and CO_3_^2−^ concentrations adopted acid-base titration. Salinity (μmol∙g^−1^) was the sum of eight common ion concentrations (K^+^, Ca^2+^, Na^+^, Mg^2+^, Cl^-^, SO_4_^2-^, CO_3_^2-^, HCO_3_^-^).

For chlorophyll *a* (Chl *a*, μg∙g^−1^) and scytonemin (Scyt., unit∙mg^−1^ fresh weight) determinations, the crust samples were directly ground in acetone, and the extractions were also conducted at 4°C in darkness overnight. The contents of Chl *a* and Scyt. were determined spectrophotometrically with the trichromatic equations of Garcia-Pichel and Castenholz (Garciapichel and Castenholz, 1991) and Garcia-Pichel et al (Garcia-Pichel et al., 1992). The bacteriochlorophyll *a* (BChl *a*, μg∙g^−1^) was determined by previous methods (Spring et al., 2009). The extracellular polysaccharide (EPS, mg∙g^−1^) were determinated using phenol-sulphuric acid assay with reference Dubois’s method (Dubois et al., 1956).

Total organic carbon (TOC), total nitrogen (TN) and total phosphorus (TP) were measured referring to previous study (Lan et al., 2014), and expressed as g∙kg^−1^ biocrusts.

The soil alkaline protease (ALPT, mg∙d^−1^∙g^−1^), soil alkaline phosphatase (ALP, μmol∙d^−1^∙g^−1^), and soil-β-glucosidase (β-GC, μmol∙d^−1^∙g^−1^) activities were determined by extracellular enzyme activity kit procedures (Suzhou Comin Biotechnology Co., Ltd., Suzhou, China). The net photosynthetic rate (Pn, μmol carbon dioxide (CO_2_) m^−2^ s^−1^) and respiratory rate (R, μmol CO_2_ m^−2^ s^−1^) were measured at temperature of 25°C and photosynthetically active radiation of 250 μE m^−2^ s^−1^ by the Soil Carbon Release Rate Measuring Device (Beijing Yaxinliyi Science and Technology Co., Ltd., Beijing, China).

**The DNA extraction**

Total genomic DNA was extracted from biocrust samples using the PowerSoil ®DNA Isolation Kit (Mo Bio, Carlsbad, CA USA). The handbook of PowerSoil ®DNA Isolation Kit could be found in <https://www.qiagen.com/>. All sequencing reads generated in this study are publicly available through the SRA database under accession number PRJNA640847. The environmental dataset can be found in FigShare (https://doi.org/10.6084/m9.figshare.13172411.v1).

**Supplementary reference**

Dubois, M., Gilles, K.A., Hamilton, J.K., Rebers, P.A., and Smith, F. (1956). COLORIMETRIC METHOD FOR DETERMINATION OF SUGARS AND RELATED SUBSTANCES. *Analytical Chemistry* 28(3)**,** 350-356. doi: 10.1021/ac60111a017.

Garcia-Pichel, F., Sherry, N.D., and Castenholz, R.W. (1992). Evidence for an ultraviolet sunscreen role of the extracellular pigment scytonemin in the terrestrial cyanobacterium Chlorogloeopsis sp. *Photochemistry and photobiology* 56(1)**,** 17-23. doi: 10.1111/j.1751-1097.1992.tb09596.x.

Garciapichel, F., and Castenholz, R.W. (1991). CHARACTERIZATION AND BIOLOGICAL IMPLICATIONS OF SCYTONEMIN, A CYANOBACTERIAL SHEATH PIGMENT. *Journal of Phycology* 27(3)**,** 395-409. doi: 10.1111/j.0022-3646.1991.00395.x.

Lan, S., Wu, L., Zhang, D., and Hu, C. (2012). Successional stages of biological soil crusts and their microstructure variability in Shapotou region (China). *Environmental Earth Sciences* 65(1)**,** 77-88. doi: 10.1007/s12665-011-1066-0.

Lan, S., Zhang, Q., Wu, L., Liu, Y., Zhang, D., and Hu, C. (2014). Artificially Accelerating the Reversal of Desertification: Cyanobacterial Inoculation Facilitates the Succession of Vegetation Communities. *Environmental Science & Technology* 48(1)**,** 307-315. doi: 10.1021/es403785j.

O'Dwyer, J.P., Kembel, S.W., and Sharpton, T.J. (2015). Backbones of evolutionary history test biodiversity theory for microbes. *Proceedings of the National Academy of Sciences of the United States of America* 112(27)**,** 8356-8361. doi: 10.1073/pnas.1419341112.

Spring, S., Luensdorf, H., Fuchs, B.M., and Tindall, B.J. (2009). The Photosynthetic Apparatus and Its Regulation in the Aerobic Gammaproteobacterium Congregibacter litoralis gen. nov., sp nov. *Plos One* 4(3). doi: 10.1371/journal.pone.0004866.
